# Virtual medicine: medical AI in human health and diseases

**DOI:** 10.1016/j.mmr.2026.100012

**Published:** 2026-04-04

**Authors:** Chen Zhang, Fu-Xiao Wang, Xuan Tang, Ji-Long Li, Ning Ding, Yang Hong, Pei-Ran Song, Long Bai, Jia-Can Su

**Affiliations:** a Organoid Research Center, Institute of Translational Medicine, Shanghai University, Shanghai 200444, China; b MedEng-X Institutes, Shanghai University, Shanghai 200444, China; c School of Medicine, Shanghai University, Shanghai 200444, China; d National Center for Translational Medicine (Shanghai) SHU Branch, Shanghai University, Shanghai 200444, China; e Wenzhou Institute of Shanghai University, Wenzhou 325000, Zhejiang, China; f Department of Orthopaedics, Xinhua Hospital Affiliated to Shanghai Jiao Tong University School of Medicine, Shanghai 200092, China

**Keywords:** Medical AI, Clinical decision support, Virtual Medicine, Precision medicine, Large language models

## Abstract

The evolution of medicine has progressed through distinct ages: from empirical observation and evidence-based practice to the current era of precision medicine. However, traditional healthcare paradigms remain constrained by data fragmentation, scalability limits, and the overwhelming complexity of multi-omics integration. In the rapid explosion of artificial intelligence (AI), a transformative paradigm is emerging. This review introduces the concept of “Virtual Medicine”, which is defined as a comprehensive ecosystem of AI-empowered medical practice that transcends physical limitations. This review systematically summarizes the technological foundations, historical evolution, and core applications of AI in medicine, including electronic health records (EHRs) analysis, medical imaging, multimodal diagnostics, drug discovery, precision oncology, intelligent surgery, and clinical decision support systems. It further highlights the role of medical AI in health management, public health surveillance, and healthcare delivery in resource-limited settings. Special attention is given to the transformative emergence of large language models (LLMs), such as medical large language models (MedLLM) and generative pre-trained transformer (GPT) architectures, emphasizing their potential to revolutionize virtual medical interaction, clinical reasoning, and documentation. Despite these advances, significant challenges remain regarding model transparency, data bias, fairness, and patient privacy. Overcoming these limitations necessitates standardized evaluation frameworks, interpretable algorithm designs, and strengthened privacy protections. Ultimately, these efforts aim to foster a trustworthy and equitable future for virtual medicine.

## 1 Background

**Fig. 1:**
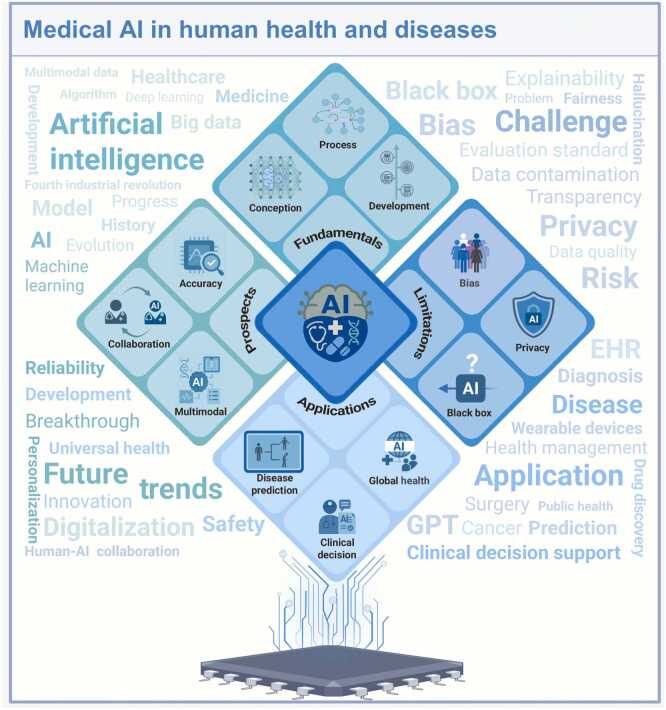
An overview of medical AI in human health and diseases. AI. Artificial intelligence; GPT. Generative pre-trained transformer; EHR. Electronic health records.

Faced with an aging global population, a growing burden of chronic diseases, and unequal access to healthcare resources, traditional healthcare systems are increasingly struggling in terms of efficiency, diagnostic accuracy, and resource allocation [1], [2]. These constraints highlight the urgency of a paradigm shift toward “Virtual Medicine”, a digitalized ecosystem that integrates advanced technologies to optimize disease prevention, diagnosis, and treatment (Fig. 1).

Over the past decades, artificial intelligence (AI) has evolved from a conceptual framework to a practical engine for medical innovation. Advances in machine learning (ML) and natural language processing have catalyzed applications in areas such as medical imaging, clinical prediction, drug development, and decision support [3]. Medical AI systems now assist in identifying subclinical lesions, triaging high-risk patients, and tailoring personalized treatment plans, surpassing conventional performance in several diagnostic tasks and accelerating clinical workflows [4], [5]. A study on diabetic retinopathy screening in Africa has achieved an automatic interpretation sensitivity of more than 97%, providing accurate diagnosis for residents under poor medical conditions [6]. Alongside these advances, critical challenges persist. Model opacity undermines interpretability and clinical trust, biased training datasets risk exacerbating health disparities, and the reliance on patient data raises profound concerns over privacy and data security [7]. These issues require rigorous validation, ethical safeguards, and systematic model governance to ensure safe, transparent, and equitable deployment [8].

Several works have discussed the role of AI in medicine. For instance, Rajpurkar et al. [3] summarized technical progress and validation studies, particularly in imaging and molecular applications, while He et al. [8] focused on the practical challenges of clinical implementation. However, these reviews are either domain-specific or limited to early implementation perspectives. In contrast, this review provides a more comprehensive synthesis across the entire clinical continuum: from disease diagnosis, clinical treatment, and surgical intervention to health management and public health. Moreover, it integrates the latest advances in large language models (LLMs), multimodal AI, and fairness frameworks, which have not been systematically covered in earlier reviews. By combining technical, clinical, and ethical perspectives, this review aims to deliver a holistic understanding of medical AI and outline future directions for safe, equitable, and effective integration into modern medicine.

## 2 Overview of medical AI

### 2.1 Fundamental concepts of AI

Analogous to the transformative impact of the electrical power system during the second industrial revolution, the advent of AI is precipitating a comparable shift in productivity and industrial organization, commonly referred to as the “fourth industrial revolution” [9]. The definition of AI, in its most fundamental sense, encompasses systems that simulate or augment human intelligence through computational or hardware-based mechanisms. Such systems can perform tasks that normally require human cognitive functions including natural language processing, visual recognition, and audio signal interpretation [10]. Distinct from traditional software, the core of AI lies in its capacity for autonomous learning, reasoning, perception, comprehension, and decision-making-features that have made its application in healthcare increasingly pervasive [11]. When confronted with vast pathological datasets and complex clinical problems, AI demonstrates formidable capabilities in data processing and decision support [12]. By learning from physical examination data and patient records, AI can efficiently extract disease-related patterns and information, thereby assisting clinicians in early disease diagnosis and treatment planning [13].

### 2.2 Historical development of medical AI

**Fig. 2:**
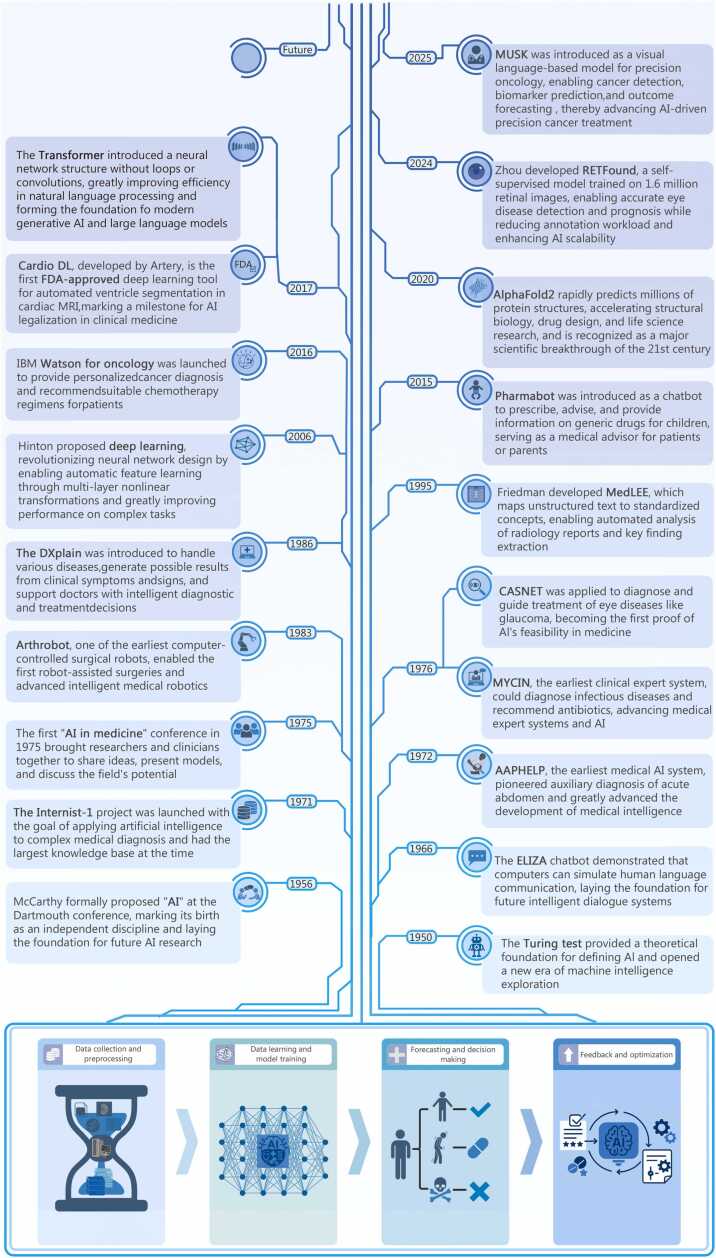
The historical development and decision-making principles of medical artificial intelligence (AI). DL. Deep learning; FDA. U.S. Food and Drug Administration; MRI. Magnetic resonance imaging; IBM. International Business Machines Corporation; MedLEE. Medical language extraction and encoding system.

The evolution of AI from a conceptual framework to a transformative technology in medicine has been marked by a series of seminal technological breakthroughs and progressive integration with clinical applications (Fig. 2). The earliest formal concept of AI can be traced back to the Dartmouth Conference in 1956, where John McCarthy often regarded as the “father of AI”, first launched the term “AI”, marking the birth of modern AI research [10]. However, explorations into AI began even earlier. In 1943, American psychologists McCulloch and mathematician Pitts proposed the mathematical model of binary neurons, inspired by the workings of biological neurons, thus laying the groundwork for neural network theory [14]. Alan Turing introduced the Turing Test in 1950, which remains a classic benchmark for assessing AI.

Building on this foundational work, AI experienced rapid development. Nine years after the dartmouth conference, the chatbot ELIZA was introduced as an early exploration in natural language processing, paving the way for future medical consultation systems [15]. Released in 1976, the MYCIN system represented the first implementation of an AI-based expert system in medicine [16]. Developed at Stanford University, MYCIN focused on diagnosing infectious diseases and selecting appropriate antibiotics. By inputting patient information into a computer, MYCIN could use logical inference to recommend optimal antibiotic regimens, showcasing the clinical potential of AI for the first time [16]. Similarly, DXplain, introduced in 1986, was another influential medical expert system based on probabilistic models [17]. It integrated information across multiple medical fields to provide broad differential diagnoses for clinical symptoms and signs, thus supporting clinicians in decision-making [17].

With the advent of the 21st century, AI technology experienced significant expansion due to breakthroughs in deep learning (DL) [18], extending its medical applications beyond diagnostic support to areas such as medical image analysis, personalized therapy, and public health management [19]. DL theory, proposed by Hinton et al. [20] in 2006, revolutionized neural network architecture by enabling automatic feature extraction through multi-layer nonlinear transformations, greatly enhancing model capacity for complex tasks.

In 2015, the pharmabot conversational chatbot was born, trained to prescribe, advise, and provide information about drugs for children. They introduced an application that can act as a medical advisor to patients or parents who are confused about generic drugs [21]. In 2017, Cardio DL, developed by Artery, became the first U.S. Food and Drug Administration (FDA)-approved DL-based medical application, automating cardiac magnetic resonance imaging (MRI) segmentation with accuracy on par with experienced clinicians [22], [23]. This regulatory milestone sets the stage for the legitimization of AI in clinical medicine. That same year, the introduction of the transformer architecture created a structure that does not require loops and convolutions, greatly improving the efficiency and performance in tasks such as Natural Language Processing (NLP), and laying the foundation for the development of modern GPT and LLMs [24]. In 2020, AlphaFold2 made a landmark breakthrough in protein structure prediction, solving the long-standing protein folding problem and accelerating the development of peptide drugs and structural biology [25]. In 2024, Zhou et al. [26] developed RETFound, which can efficiently perform eye disease detection and prognosis tasks, reduce the burden of expert annotation, and enhance the scalability and generalization of AI in retinal imaging and broader clinical applications. In 2025, the MUSK model was introduced as a visual language-based model for precision oncology [26]. MUSK is capable of performing cancer detection, molecular biomarker prediction, and predicting clinical outcomes such as recurrence, prognosis, and immunotherapy response, thereby significantly improving the accuracy and universality of AI in precision cancer treatment [27].

Over these decades, through continuous algorithmic innovation and advances in sensing technologies, AI has gradually attained sophisticated autonomous learning and decision-making capabilities, driving the development of modern medicine towards personalization and intelligence [28].

### 2.3 The basic decision-making process of medical AI

The decision-making process of AI in medicine typically encompasses 4 main stages: data collection and preprocessing, data learning and model training, prediction and decision-making, and feedback and optimization [29].

Medical data is often sourced from a variety of records, including Electronic Health Records (EHRs), medical images, genomic sequences, and real-time monitoring signals [30], [31], [32], [33]. Such data is characterized by its multi-source and diverse nature, with both structured data and unstructured data coexisting. Given the variability in data type and quality, preprocessing is crucial to enable effective AI analysis. During this phase, missing and unusual values are addressed through data cleaning, image denoising techniques are applied to reduce interference in medical imaging, normalization is achieved via standardization of units and formats, and feature engineering extracts key indicators, converting raw data into a computable form [34].

Data learning and model training constitute the core steps in transforming medical data into AI models. Using appropriate algorithms, AI systems can identify disease patterns and risk factors from preprocessed medical data. For example, in disease diagnosis, medical AI applies ML algorithms such as support vector machines (SVMs), regression analysis, or decision trees to discover relationships between clinical features and symptoms from large patient groups, thereby generating predictive outcomes for clinicians [35], [36]. In this process, datasets are divided into training and validation sets; the model repeatedly adjusts its parameters to minimize prediction error and achieve optimal fitting. Medical AI may use unsupervised and reinforcement learning to further refine its decision-making, enabling effective prediction and decision support even when encountering novel or previously unseen cases [37], [38].

Prediction and decision-making form the core of medical AI applications. Trained models infer outcomes from new data and support clinical decision-making. For instance, an AI system may analyze digital mammography images to predict the malignancy probability of a tumor [39], or anticipate a patient’s drug response based on genomic information and medical history [40]. Throughout this process, medical AI decisions incorporate confidence estimation and explainability techniques to ensure clinical reliability, and may synthesize results from multiple models or rule-based systems to deliver high-confidence, personalized recommendations [41].

Feedback and optimization are essential to the continuous improvement of AI systems. After clinical validation, differences between AI recommendations and real-world outcomes are fed back into the model, triggering retraining or parameter adjustment to iteratively refine the system. As more data accumulates, medical AI models progressively enhance their decision quality, matching more closely with actual clinical needs [42].

Through these stages, medical AI establishes a closed-loop system from raw data to clinical decision-making. The key strength lies in data-driven optimization, translating statistical patterns into actionable medical knowledge that supports clinicians in achieving greater accuracy and efficiency in diagnosis and treatment.

### 2.4 Advantages of medical AI

As the volume of medical data continues to expand, AI demonstrates multiple advantages within healthcare. Medical AI can rapidly process and analyze massive datasets, extracting hidden patterns through DL algorithms to provide a scientific basis for disease diagnosis and precision therapy [43]. The efficiency and accuracy of medical AI reduce errors associated with manual operations, thereby improving the reliability and safety of medical workflows [44]. Medical AI excels in real-time monitoring, disease progression prediction, and therapeutic optimization, offering clinicians more precise and personalized treatment support [45].

With continuous learning and self-improvement, medical AI systems refine their models through the accumulation of clinical cases, maintaining a leading edge in diagnostic and therapeutic capabilities. Unlike traditional experience-based approaches, medical AI utilizes objective clinical data, enabling rapid advancement in both diagnostic speed and accuracy. The automation and standardization afforded by medical AI allow it to assist clinicians in repetitive and highly regulated tasks such as image interpretation and laboratory result analysis, helping to reduce the burden on doctors [46].

In the context of population aging and unequal distribution of medical resources, medical AI not only alleviates the workload of healthcare professionals but also optimizes the patient care experience [47]. Through efficient data analysis, ongoing technological improvement, and process automation, medical AI is setting the stage for the future of medicine and is set to become an indispensable driving force in healthcare.

## 3 The application of medical AI in disease diagnosis

### 3.1 AI-based disease diagnosis using EHRs

EHRs are digital versions of patients’ medical histories maintained over time, including comprehensive clinical details including patient information, diagnoses, medications, treatment plans, immunizations, allergies, radiology images, and laboratory results [48]. They offer substantial value in healthcare delivery, ensuring continuity of patient care and reducing medical errors by enabling patient information sharing among healthcare providers [49].

Integrating EHRs with AI enables earlier prediction and identification of diseases that aredifficult to diagnose. Utilizing ML and NLP techniques, AI algorithms can analyze structured and unstructured data within EHRs, identifying subtle clinical patterns indicative of early-stage diseases [50]. For instance, AI algorithms using NLP on unstructured clinical notes significantly enhance early detection of sepsis, a condition known for subtle early symptoms but rapid progression. The SERA algorithm, utilizing structured and unstructured EHR data, has demonstrated a high predictive accuracy 12 h before clinical diagnosis, substantially surpassing traditional clinical assessments. This algorithm notably improved sensitivity and reduced false positives, critical factors in timely sepsis management [51].

In oncology, medical AI integrated with EHRs data facilitates continuous pan-cancer prognostication, significantly improving early cancer detection and personalized management strategies. For example, the MEDomics platform incorporates multimodal health data from EHR systems to create individualized patient profiles [52]. These profiles have successfully predicted hospitalizations and emergency visits in cancer patients by analyzing structured clinical variables and unstructured clinical notes using NLP. This approach has led to clear improvements in patient outcomes, including reduced emergency visits and hospital admissions, through earlier identification and management of complications [53].

Pediatric medicine also benefits substantially from AI-enhanced EHR analysis. AI algorithms have shown considerable effectiveness in diagnosing complex pediatric conditions, where early clinical signs might not be overtly discernible. These models integrate multiple clinical variables, enhancing diagnostic precision and facilitating earlier therapeutic interventions, which can markedly improve long-term pediatric health outcomes [54].

Despite these advances, using EHRs data for predictive analytics encounters inherent challenges. Biases originating from the healthcare process, such as variations in clinician documentation practices, incomplete records, and inconsistent coding of clinical information, pose significant obstacles [55]. Additionally, missing data points and inaccuracies in unstructured notes can reduce predictive reliability [56]. To mitigate these issues, employing advanced data optimization techniques, including robust data imputation methods, NLP-driven error correction, and standardized data entry protocols, is crucial. The rigorous validation of EHRs-derived predictive models across multiple institutions further ensures broad applicability and reliability.

Future directions should emphasize enhancing data governance, adopting standardized ontologies, and fostering collaborative, multi-institutional data sharing. These strategies will significantly refine EHRs’ data quality, advancing AI-driven predictive capabilities and fostering robust, real-time clinical decision support systems [57].

### 3.2 AI-based disease diagnosis using medical imaging

During diagnosis and treatment, clinicians rely heavily on imaging modalities such as X-rays, MRI, computed tomography (CT), and electrocardiograms (ECGs) to visualize internal anatomical and physiological changes [58]. These images provide crucial, intuitive insights into disease progression. However, the large amount of imaging data, combined with subtle early-stage anomalies that may be missed by human observers, presents significant diagnostic challenges and workload burdens.

To address these limitations, AI technologies, especially convolutional neural networks (CNNs) and advanced image preprocessing, are being applied to medical imaging [59]. These algorithms exhibit high efficiency, sensitivity, and standardization, often matching or even surpassing expert radiologists in diagnostic performance. For instance, in breast cancer screening, a DL model using mammography and digital breast tomosynthesis outperformed 5 full-time breast imaging specialists, boosting sensitivity by 14% [60]. Similarly, AI-enhanced CT analysis has demonstrated high accuracy in diagnosing COVID-19 pneumonia, even in early or atypical cases, and provided predictive insights into disease severity and progression [61].

Medical AI is also revolutionizing cardiovascular diagnostics. In ECG analysis, DL models trained on 90,000 ECG-echocardiogram pairs identified asymptomatic left ventricular dysfunction, equaling the accuracy of standard echocardiographic methods [62]. Likewise, Cardiac magnetic resonance imaging (CMR) has been accelerated by medical AI to automatically screen for and diagnose multiple cardiovascular conditions with a performance that in some cases exceeds that of experienced cardiologists [63].

These advancements are not standalone, and hybrid medical AI workflows are proving especially effective. For example, decision-referral systems in breast cancer screening allow AI to automatically triage high-certainty cases, while uncertain evaluations are directed to radiologists. This collaboration improved both sensitivity and specificity beyond what either could achieve alone [64]. Moreover, AI can also repair artifacts and damage in defective three-dimensional (3D) biomedical imaging and reconstruct it, truly restoring tissue structure and cell distribution, greatly improving the spatial continuity and quantitative analysis accuracy of 3D reconstruction [65].

The above examples demonstrate the significant advantages of medical AI in imaging diagnostics. It can efficiently process large-scale datasets, enhance the sensitivity and accuracy of early abnormality detection, and achieve diagnostic performance comparable to or even surpassing that of human experts across multiple disease contexts. In addition, AI systems are capable of repairing defective images, thereby improving diagnostic reliability. However, notable limitations remain. The training of AI models for image recognition relies heavily on large, high-quality annotated datasets, and inconsistencies in annotation standards hinder clinical translation and broader deployment of medical AI.

### 3.3 AI-based disease diagnosis using multimodal data

Relying on one type of data, whether textual or visual, to diagnose complex diseases is not sufficient for comprehensive healthcare solutions. Medical diagnoses are inherently multifactorial and often require the integration of various data sources for accurate predictions. Therefore, multimodal medical AI models, which combine information from multiple sources including clinical laboratory results, medical imaging, genomics, and patient demographics, provide a more complete method for disease diagnosis and prediction [66], [67].

Multimodal AI has also demonstrated significant value in the diagnosis of dementia. Differentiating between various types of dementia, such as Alzheimer’s, vascular dementia, and Lewy body dementia, remains a major challenge due to the overlapping symptoms. Multimodal medical AI models with the ability to analyze neuroimaging, genetic markers, neuropsychological testing, and clinical history can diagnose multiple causes of dementia. For example, models trained using multimodal datasets of neuroimaging and clinical datasets can accurately differentiate between Alzheimer’s disease and vascular dementia, showing significant improvement over single-modality approaches [68]. Additionally, the Medical Multimodal Multitask Foundation Model (M3FM) can be used for lung cancer screening, and clinical teams apply M3FM for comprehensive risk assessment. The model integrates CT images, smoking records, family cancer history, and laboratory test results to assess lung cancer risk [69]. Furthermore, the M3FM extends to other related tasks, such as cardiovascular disease (CVD) mortality risk prediction and incidental finding detection [69].

The advantage of such multimodal models is that they can adapt to diverse data sets, thereby providing more accurate and personalized predictions [70]. While traditional methods may overlook key features due to the limitations of unimodal inputs, multimodal models mimic the diagnostic methods of human clinicians. Multimodal medical AI models can not only improve diagnostic accuracy but also assist doctors in making better decisions [71]. Improvements in these models mark the future of medical diagnostics. Their ability to combine diverse datasets allows for a more robust and individualized understanding of health, thus advancing precision medicine.

## 4 The applications of medical AI in disease treatment

### 4.1 AI-enabled drug discovery and repurposing

AI plays a crucial role in predicting disease occurrence and enhancing therapeutic strategies. Drug therapy remains a foundation of clinical treatment, yet identifying effective drugs for complex diseases is often slow and expensive [72]. Therefore, accelerating the drug discovery process is an urgent need. One of the most influential areas where AI contributes is in target screening and early drug discovery. Medical AI can process large-scale biological data to identify disease-relevant targets and predict ligand-receptor interactions [73]. DL algorithms are used to evaluate molecular properties and binding affinities, improving the efficiency of virtual screening. Additionally, AlphaFold has revolutionized protein structure prediction by generating high-confidence 3D models of proteins, including those without experimental structures [74]. These predicted structures allow researchers to perform structure-based drug design at scale, supporting faster identification of candidate molecules and reducing reliance on expensive laboratory-based assays.

Medical AI also facilitates the discovery of new structures of antibiotics, addressing the global issue of antibiotic resistance. Traditional antibiotics are losing efficacy due to widespread overuse, and new structural classes are urgently needed [75]. Recent studies have applied graph neural networks (GNNs) to explore chemical spaces containing millions of compounds [76], [77]. These models identify substructures associated with antimicrobial activity and low cytotoxicity. In a notable example, explainable AI was used to screen over 12 million compounds and predict new antibiotic candidates. The medical AI successfully identified structural classes selective against drug-resistant pathogens, demonstrating activity in both in vitro and in vivo models [78].

Another critical application of medical AI lies in drug repurposing. Repurposing existing drugs for new indications can reduce development time and cost, offering a viable solution for diseases with limited therapeutic options [79]. The TxGNN model demonstrates this approach, using a knowledge graph to learn relationships between drugs, targets, and diseases. It supports zero-shot predictions and can identify drug candidates for both treated and untreated diseases. In addition, the model is able to generate interpretable results that are consistent with expert clinical decisions [80].

In short, medical AI is transforming drug development and disease treatment. It accelerates the discovery of targets and candidate molecules, speeds up the development of new antibiotics, and streamlines effective drug repurposing processes.

### 4.2 AI-enabled precision medicine

Precision medicine is an evolving healthcare approach where therapies are customized for each person according to the specific features of their condition, including molecular profiles [81]. This field has become increasingly significant in oncology, where the complexity of cancer demands personalized therapies to improve outcomes. The core principle of precision medicine is to customize therapy guided by an individual’s genomic profile and the unique properties of their tumor, and other factors, thus optimizing treatment effectiveness and minimizing side effects [82]. In cancer care, this approach holds the capacity to revolutionize how oncologists diagnose and treat the disease by focusing on the molecular and genetic basis of cancer rather than the traditional one-size-fits-all model. However, the successful application of precision medicine hinges on the integration and analysis of massive and complex datasets from multiple domains, such as genomics, transcriptomics, proteomics, and metabolomics [83], [84]. These omics technologies, clinical information, and molecular biomarkers can provide a comprehensive picture of the tumor and its microenvironment and provide important information about the disease’s response to treatment.

Manual evaluation of these datasets is time-consuming, error-prone, and expensive. The lack of standardized procedures can also cause inconsistent results and hinder the scalability of precision medicine. This is where medical AI steps in, offering remarkable capabilities for processing and analyzing large datasets. AI’s ability to discover connections between data from multiple perspectives can accelerate the development of platforms that support not only diagnostic confidence but also personalized treatment approaches. DL and ML algorithms are particularly good at processing the massive multi-omics datasets generated by cancer research [85].

AI medical models show promise in early cancer detection using multi-omics analysis. The CancerSEEK test combines genomic and protein biomarkers from blood tests to detect a variety of cancers. The technology is highly sensitive and can be used to detect ovarian, liver, and pancreatic cancers, sometimes even before symptoms appear [86]. Medical AI is also being used to enhance radiogenomics, combining radiological imaging and genomic data to enhance cancer identification and prediction of disease progression [87]. The application of medical AI in multi-omics datasets is not limited to diagnostic prediction. Patient responses to treatment can be predicted by AI systems, allowing the best treatment to be selected. Medical AI has been used to predict immunotherapy responses based on proteomic datasets, enabling personalized immunotherapy treatment [88].

### 4.3 AI-enhanced surgery task

Surgery is the foundation of modern medicine. However, as the complexity and risks of surgery increase, it is essential to improve surgical precision and achieve optimal efficacy and safety. In recent years, AI has increasingly penetrated the field of surgery, striving to revolutionize medical practice [89].

AI’s involvement in surgery is multifaceted, beginning with its ability to assess surgical competence. Studies have shown that a surgeon’s skill level directly reflects patient outcomes [90], [91]. Medical AI can assess surgical outcomes in real time based on robotic surgery data [92]. This unbiased assessment is critical, as traditional evaluations often rely on expert assessments, which are both subjective and variable. ML algorithms can efficiently analyze large amounts of surgical data, extracting details that might be overlooked, enhancing clinical outcome predictions, and informing postoperative care choices [93]. By reviewing surgical techniques, postoperative outcomes, and recovery, medical AI can support future treatment planning, resulting in more personalized care for patients.

Beyond assessment, AI can also play a role directly in surgical procedures, through the use of robotics. The da Vinci Surgical System stands out as a widely implemented technology in this field, which has been adopted across numerous surgical disciplines [94]. These robotic systems allow surgeons to perform minimally invasive surgeries with greater precision, using robotic arms controlled from a console [95]. AI-based systems can help doctors fine-tune procedures, provide real-time feedback, and improve accuracy [96]. This smart assistance is particularly beneficial in complex procedures where precision is critical, such as in prostatectomies or cardiovascular surgeries [97].

Medical AI will be used to enhance training. By reviewing surgeries, medical AI can assess surgeons’ technical proficiency, helping them improve their skills. In some systems, DL is used to identify and correct technical errors such as improper suturing techniques during live surgeries. These systems not only promote continuous learning but also ensure that surgeons maintain proficiency throughout their careers [98].

Future innovations could lead to fully autonomous robotic surgeries, where AI moves beyond mere assistance to perform certain tasks independently, guided by vast data and ML algorithms. However, the integration of medical AI in surgery should be accompanied by rigorous evaluation frameworks, such as the IDEAL framework, to ensure safety, effectiveness, and ethical application [99].

In conclusion, medical AI is transforming surgery by enhancing precision, improving training, and evaluating surgeon performance. Its integration into robotics is transforming surgical intervention with better patient outcomes as well as a more efficient surgical method [100].

## 5 The applications of medical AI in health management and disease prevention

### 5.1 AI-powered health management

AI not only serves a key function in the identification and management of illnesses but also enables continuous health monitoring beyond the clinic [101]. By analyzing patient behavior, heart rate or audio data collected from wearable devices, medical AI can detect hidden disease-related patterns, enabling ongoing disease monitoring and management [102]. In the following, we will describe how wearable technology combined with medical AI is transforming predictive health monitoring through multimodal data collection and intelligent analysis.

Wearable devices vary widely in form and function, ranging from wrist-worn sensors and smartwatches to full-body suits equipped with multiple inertial measurement units (IMUs). These devices can continuously capture physiological and biomechanical signals, including heart rate, skin temperature, movement kinematics, and electrodermal activity. For instance, in a study of Friedreich’s ataxia (FA), a full-body motion capture suit embedded with 17 inertial sensors was used to monitor patients during clinical assessments such as the 8-meter walk and 9-hole peg tests [103]. These digital behavioral features, when analyzed by ML algorithms, allowed for the prediction of disease progression with significantly higher precision than traditional clinical scales. In another example involving Duchenne muscular dystrophy (DMD), researchers used a wearable 17-sensor bodysuit to capture daily-life movement data over a 12-month period. ML models were then applied to define behavioral “fingerprints” that accurately tracked disease progression and therapeutic responses [104]. These digital biomarkers offered a more objective and personalized method of assessment compared with conventional clinical tests.

A particularly promising area is the use of medical AI to detect and monitor Parkinson’s disease (PD) through nocturnal breathing signals. A study has demonstrated that a single night of breathing data can be analyzed using medical AI models to detect PD with high accuracy and estimate disease severity based on the Movement Disorder Society Unified Parkinson’s Disease Rating Scale (MDS-UPDRS) [105]. Notably, these data can be collected either through wearable breathing belts or non-contact devices mounted on walls, which use low-power radio waves to measure chest and abdominal motion. This contactless setup allows for long-term monitoring without disrupting the patient’s sleep environment, promoting better adherence and improved data quality [106], [107].

As wearable technologies continue to evolve, the focus is shifting toward highly integrated, inconspicuous, and user-friendly designs [108], [109]. Future systems are expected to achieve “invisible” wearability, minimizing user discomfort while maximizing data quality. This trend will further enable AI to utilize continuous, real-world data for personalized, predictive, and preventive healthcare, opening new frontiers in digital medicine.

### 5.2 AI-powered public health

AI is increasingly applied to both personal and public health, particularly in infectious disease control [110]. This application of medical AI expands beyond individual diagnostics and treatments to include a broader range involving population-wide surveillance, risk management, and healthcare resource optimization.

In the early stages of disease outbreaks, AI technologies significantly improve surveillance and early-warning capabilities [111]. For instance, HealthMap utilizes NLP and ML algorithms to scan global web content continuously, detecting and classifying disease-related information [112]. This AI-driven system was notably successful in identifying the emergence of COVID-19 through early detection of pneumonia clusters of unknown etiology, providing crucial early alerts to public health authorities [113]. Once a disease outbreak is identified, medical AI also plays a critical role in population risk assessment. AI-powered predictive analytics, such as ML models using syndromic surveillance data, effectively assess risk and predict the severity of infections like dengue fever and COVID-19, enabling targeted public health interventions and optimized resource allocation [114].

Additionally, medical AI accelerates the development of antiviral drugs through structural modeling and simulation. ML methodologies, including graph neural networks and generative models, have been instrumental in virtually screening extensive compound libraries [115]. These approaches enable the rapid identification of potential antiviral candidates during pandemics such as COVID-19 [110]. These techniques allow researchers to rapidly target new molecular structures, shortening the timeline from discovery to clinical testing.

In conclusion, medical AI can enhance various areas of public health, including disease surveillance, risk assessment, antiviral drug development, and resource allocation [116], significantly transforming global capacity for health emergency preparedness and intervention.

### 5.3 AI-powered medical assistance

In many low- and middle-income countries (LMICs), limited medical resources significantly hinder the ability to deliver adequate healthcare. These limitations manifest as insufficient funding, outdated medical infrastructure, shortages of healthcare professionals, and limited access to comprehensive health data necessary for effective patient management [117].

To address these constraints, traditional models of sending medical teams from high-income countries (HICs) have often been adopted. However, such approaches are unsustainable, costly, and typically short-term solutions. Conversely, AI-assisted remote medical services have emerged as more viable, sustainable, and efficient alternatives, using advanced healthcare expertise from developed nations without physically relocating healthcare professionals [118].

The effectiveness of medical AI in LMIC healthcare contexts has been demonstrated in several practical implementations. A clinical validation study in Zambia exemplifies the impact of AI for screening diabetic retinopathy, a major cause of preventable blindness [6]. In this study, a DL-based AI model initially trained on data from a high-income setting (Singapore) was applied to Zambia’s population-based diabetic retinopathy screening program. The medical AI demonstrated robust diagnostic performance, with a sensitivity of 92.25% and a specificity of 89.04% for detecting referable diabetic retinopathy. It was particularly effective in identifying vision-threatening cases, achieving a sensitivity of 99.42%. Such outcomes highlight AI’s potential for large-scale implementation even in resource-constrained African regions, significantly contributing to early diagnosis and prevention of diabetic blindness.

Moreover, the integration of AI in antenatal care through digital health registries has also illustrated significant benefits in LMICs. For example, in Palestine, a controlled trial of an eRegistry incorporating clinical decision support significantly improved adherence to guidelines for screening and managing key antenatal conditions such as anemia, hypertension, and diabetes, as compared with traditional paper-based systems [119]. Although this did not immediately translate into improved health outcomes, the improved process adherence clearly demonstrated the potential of digital clinical decision-support systems to enhance the quality and consistency of healthcare services in LMICs, aligning closely with World Health Organization (WHO) guidelines.

Despite these promising applications, challenges remain when transferring medical AI technologies developed in high-income countries to LMIC contexts. Research conducted in Vietnam involving the use of a United Kingdom (UK)-developed medical AI model for COVID-19 triage illustrated this concern [117]. While this medical AI model initially showed strong performance in its original UK hospital setting, direct application to Vietnamese hospitals without adjustments led to significantly reduced performance [117]. Factors such as differences in population characteristics, variations in clinical practice, and differences in healthcare infrastructure were significant barriers to effective generalization of the model. This emphasizes the necessity of tailored AI solutions adapted to the specific contexts and resources of LMICs rather than a direct application of models developed in distinctly different healthcare environments.

An additional systematic scoping review reinforced these observations, noting substantial barriers to successful medical AI adoption in LMICs, including limited availability of high-quality data, mixed impacts on workflow integration, poor user-friendliness, and insufficient adaptation to local contexts [120]. This underscores that the successful implementation of medical AI in LMIC healthcare systems requires thorough contextualization and careful consideration of local data quality, infrastructure capabilities, and human factors.

## 6 Large language models in healthcare

The AI models mentioned above have performed well in the medical field, but most current medical AI systems usually rely on data labeled in specific fields for training and focus on solving specific tasks. This specialized training model limits the model’s application flexibility in cross-domain or general medical scenarios, making it difficult to generalize and deploy on a large scale in actual clinical environments.

### 6.1 GPT in healthcare

Unlike AI systems that work in fixed fields, GPT is gradually emerging as a general model. GPT is a LLM that uses massive text data for self-supervised learning pre-training. It can learn the intrinsic patterns and knowledge of language by training on a large-scale unlabeled text corpus, and then adapt to specific tasks through fine-tuning or directly apply to multiple tasks without fine-tuning. Its core technical route is based on the self-attention mechanism and transformer architecture, which enables it to flexibly process and generate coherent text information. This general and flexible training method is significantly different from traditional medical-specific AI systems and is not limited by structured data and fixed tasks [121].

Distinct from other medical AI systems that require certain professional knowledge to be used effectively, GPT is more oriented to the general public, greatly reducing the threshold for users to obtain information. This is due to its interactive, conversational question-and-answer method. Users do not need to master complex medical terms or have a medical background. They can interact with the GPT model through simple natural language to obtain relevant health advice, medical information, or answers to questions [122].

Nowadays, the range of applications of GPT in the medical field has expanded quickly and become more advanced [123]. A large number of studies have verified the application effect and potential of GPT in various scenarios such as clinical text summary generation, medical record analysis, doctor-patient communication assistance, and disease diagnosis decision support [124], [125], [126]. For example, in the clinical text summary task, studies have shown that with the assistance of the GPT model, the completeness and accuracy of clinical text summaries can reach or even exceed the level of medical experts, significantly improving the efficiency of medical documents [127], [128]. In addition, GPT has also shown excellent results in processing medical records in different languages. For example, in a medical record analysis study involving English, Spanish, and Italian, GPT-4 achieved an overall accuracy of 79% for 14 medical questions, demonstrating its great potential for automated analysis of multilingual medical texts [129]. At the same time, in open clinical management reasoning tasks, doctors using GPT assistance performed significantly better. A randomized controlled trial found that compared with traditional resources, GPT-4 assisted doctors’ clinical decision-making scores increased by an average of 6.5% (*P<*0.001), improving multiple capabilities such as diagnosis, management, and situational judgment [130]. Recently, the DeepSeek model developed by a Chinese team achieved an accuracy rate of 89.2% in the United States medical licensing examination (USMLE), exceeding GPT-4 (84.5%), and in terms of the completeness of diagnostic steps, DeepSeek scored 4.7 points, higher than GPT-4’s 4.2 points. In addition, DeepSeek also performed very well in the diagnosis task of 125 standardized patient cases, especially in the treatment recommendation task, where its performance was comparable to or even better than the GPT-4o model [131]. These results further demonstrate that the performance of open source models such as DeepSeek in clinical decision-making tasks has reached or even exceeded the most advanced proprietary large models [132].

### 6.2 MedLLM in healthcare

MedLLM is developed based on LLM, and through fine-tuning it in the medical field and optimizing the underlying model, it can better play a role in clinical practice [133]. It is pre-trained in multiple stages through medical literature and EHRs data, and is specially optimized to enhance the diagnostic reasoning process similar to that of doctors’ practice. The model uses self-guided learning and unified preference alignment strategies, closely aligns with clinical standards and hierarchical structures such as International Classification of Diseases (ICD-10) classification, and demonstrates a strong ability to diagnose diseases across multiple professional fields. Likewise, BiomedGPT is a general visual-language basic model that focuses on processing medical multimodal data. It performs well in medical imaging report generation and imaging question-and-answer tasks, and is particularly suitable for medical imaging analysis [134]. In addition, the AI consultation system (SSPEC) customized for the hospital reflects the outstanding contribution of LLM in reducing the burden of nurses and improving the doctor-patient communication experience [135]. In another study, the human papilloma virus (HPV) vaccination chatbot intervention for parents of junior high school girls significantly increased the vaccination rate, with the vaccination or appointment rate in the intervention group reaching 7.1%, while the control group was only 1.8%, and significantly increased the frequency of consultation between parents and medical professionals (49.1% vs. 17.6%, *P<*0.001), reflecting the potential of LLM in promoting public health [136].

At present, more and more LLMs in specific fields have been launched (Table 1) [133], [137], [138], [139], [140], [141], [142], [143], [144], [145], [146], [147], [148], [149], [150], such as organoids-GPT (O-GPT), a vertical large model focusing on organoid research. Organoids are “mini organs” with functions formed in 3D culture in vitro through the self-organization of stem cells, and play an important role in drug development, disease research, and regenerative medicine [151]. The release of O-GPT remedies the lack of application of vertical large models in the organoids research, providing researchers with intelligent knowledge support and efficient research tools. The advent of LLMs has significantly enhanced the efficiency of related research and promoted the in-depth application and innovation of AI technology.

**Table 1:** The latest medical large language models.

**Year**	**Name**	**Training data**	**Evaluation data**	**Application**	**Clinical relevance**	**Shortage**	**Reference**
2023	GatorTronGPT	Real clinical text	Medical NLP benchmark tasks	Human-like clinical text generation	Medium	Lack of clinical safety verification	[137]
2023	Med-MLLM	CXR, CT, PubMed, MIMIC-CXR/III	COVID-19 multimodal dataset	Rapid multimodal disease support	High	Limited clinical interpretability	[138]
2024	DeepDR-LLM	NDSP	CPSSDRE, CPSSDRM	Diabetes/retinopathy screening assistant	High	Narrow scope of application	[139]
2024	PathChat	Pathological images	Pathological cases	Pathology vision-language AI assistant	Medium	Insufficient independent diagnostic capabilities	[140]
2024	MEDIC	Pharmacist labeling instructions	Pharmacy real environment test	LLM prevents pharmacy errors	High	Limited applicability to complex drug combinations	[141]
2024	OncoLLM	Oncology patient EHR	Doctor’s proofreading	Automated clinical trial matching	Medium	Lower accuracy than doctors	[142]
2025	BrainGPT	Brain CT images	CQ500 dataset	3D brain CT reporting	Medium	Applicability limitations	[143]
2025	MedFound-DX-PA	MedCorpus, MedDX-FT	MedDX-Bench	Generalist disease diagnosis LLM	Medium	High computing power consumption	[133]
2025	Med-PaLM2	MultiMedQA	MultiMedQA	Expert-level medical Q&A	High	Occasional hallucinations and wrong answers	[144]
2025	COMPOSER-LLM	EHR from UCSD	Patient visit rate	Early sepsis prediction support	High	Strong reliance on EHR integrity	[145]
2025	Woollie	Real-world oncology data	Real-world oncology data	Cancer progression prediction LLM	Medium	Training data comes from a single source	[146]
2025	MMedIns-Llama 3	MedS-Ins	MedS-Bench	Versatile clinical medical LLM	Medium	Lack of real hospital application verification	[147]
2025	MedFound	MedCorpus	MedDX-Bench	Generalist diagnostic reasoning LLM	High	High computing power consumption	[148]
2025	EyeCLIP	Multimodal ophthalmic imaging	APTOS2019, MESSIDOR2	Multimodal ophthalmology disease diagnosis	High	Data mainly comes from Chinese patients	[149]
2025	ThyGPT	Ultrasound imaging of thyroid nodules	Diagnostic accuracy	Interactive thyroid nodule diagnosis	High	Insufficient identification of disease subtypes	[150]

3D. Three-dimensional; AI. Artificial intelligence; COVID-19. Coronavirus disease 2019; CT. Computed tomography; CXR. Chest X-ray; EHR. Electronic health record; LLM. Large language model; MLLM. Multimodal large language model; NDSP. Nicheng Diabetes Screening Project; NLP. Natural language processing; PA. Patient assessment; Q&A. Question and answering; UCSD. University of California San Diego

**Fig. 3:**
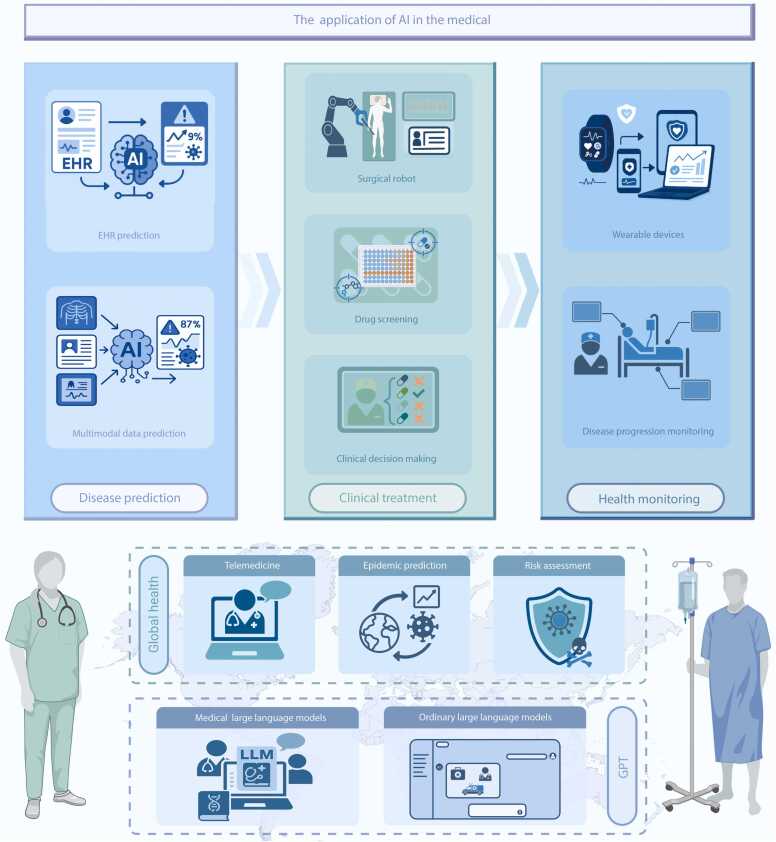
The application of medical artificial intelligence (AI). EHR. Electronic health records; GPT. Generative pre-trained transformer; LLM. Large language model.

With the continuous breakthroughs in technologies such as DL, multimodal fusion, and large models, AI is being applied to various fields of medicine, whether it is disease screening, diagnosis, treatment decision-making, or health management, public health, drug research and development, etc., it has shown unprecedented innovative vitality (Fig. 3).

### 6.3 Limitations of LLMs

Although LLMs have potential in the medical field, their existing problems cannot be ignored. LLMs have obvious racial, income, and gender biases in the medical decision-making process, such as recommending different medical interventions for different socioeconomic groups. This bias may worsen the unfair distribution of medical resources [152], [153]. In addition, although LLMs perform well on static medical record data, their performance in actual patient conversations and dynamic clinical decision-making scenarios has declined significantly [154].

LLM also faces the risk of data contamination. Alber et al. [155] have shown that only a very small amount of erroneous information (0.001%) is enough to mislead LLM’s performance in medical tasks, thus posing a potential threat to patient health. Furthermore, LLM generally has the problem of “hallucination”, that is, generating information that does not match the facts, which is particularly dangerous in the medical field [156].

To avoid these problems, standardized evaluation systems such as TRIPOD-LLM and CRAFT-MD reporting specifications have been proposed and gradually widely adopted [157]. These standardized reports provide clear evaluation criteria, including requirements in multiple aspects such as model transparency, data quality, bias identification, and clinical applicability [158]. Based on these evaluations, the current mainstream LLM still needs to be further optimized in actual clinical applications to ensure its safety, fairness, and reliability.

Despite LLM shows great potential in the medical field, in order to realize its true clinical value, it remains important to monitor the bias, data pollution, and hallucination problems in the model, and promote its safety through strict and standardized evaluation standards.

## 7 Challenges and limitations of medical AI

**Fig. 4:**
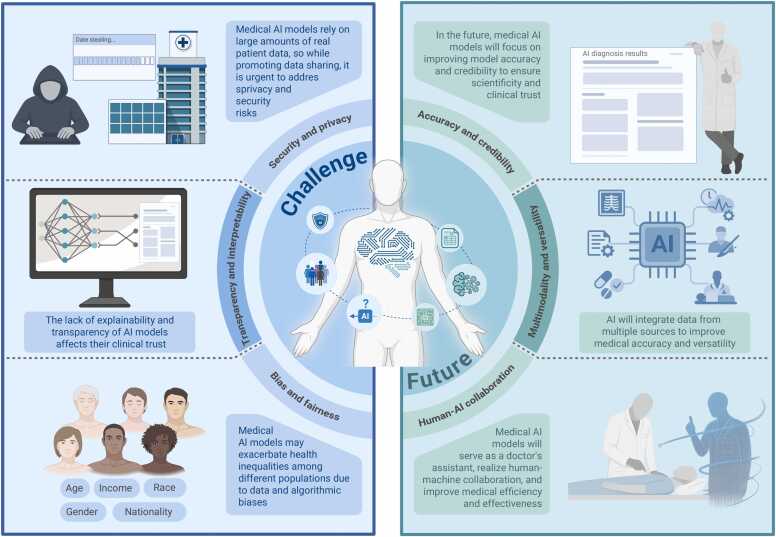
Current challenges and future prospects of medical artificial intelligence (AI).

While medical AI has significantly accelerated the progress of modern medicine, a number of challenges have emerged as a result of algorithmic limitations, incomplete data, and insufficient regulatory frameworks. The most notable concerns include bias and fairness, algorithmic interpretability and transparency, as well as data security and privacy [158], [159] (Fig. 4).

### 7.1 Bias in medical AI

Bias in medical AI refers to systematic unfairness that arises during diagnosis, prediction, or decision-making processes due to factors such as data imbalance, model design, or broader social structures. This bias often manifests as significant variations in predictive performance across different racial, gender, age, or socioeconomic groups [160]. As a result, certain populations may receive lower-quality care, further worsening existing health disparities. For example, studies have revealed that mainstream health risk assessment algorithms in the United States often underestimate the health risks of Black patients during resource allocation [161], [162]. This occurs because these algorithms incorporate “healthcare costs” as a proxy variable, which, in turn, undervalues the needs of minority patients, even when they have more severe conditions, leading to insufficient access to medical support [163]. The primary cause lies in structural inequalities embedded in healthcare spending. Due to multiple socioeconomic barriers, minority groups such as Black patients typically have reduced access to healthcare resources. During training, AI algorithms “learn” and further reinforce or intensify these inequities [164]. Beyond racial bias, gender bias is also serious in medical AI. Many algorithms are developed without adequate consideration of gender-specific factors, resulting in the underrepresentation or misdiagnosis of women, lesbian, gay, bisexual, transgender, and queer/questioning (LGBTQ) individuals, and transgender patients [165]. For instance, in medical imaging and psychiatric AI applications, the rate of missed diagnoses is higher for women and minority groups, and recommendations are often less accurate, directly impacting the fairness and effectiveness of healthcare [166].

To address these challenges, the medical community and AI developers are actively exploring various solutions. There is a need to improve the diversity and representativeness of datasets at the source, ensuring adequate inclusion of minority populations, different genders, and varied social backgrounds [167]. In recent years, organizations such as the National Institutes of Health (NIH) have promoted the development of multicenter, multiethnic high-quality medical datasets, which help alleviate data bias [163]. Additionally, algorithm development should focus on the appropriate selection of objective functions, avoiding the use of easily accessible but structurally flawed proxy variables in place of actual health needs. During model evaluation and deployment, fairness assessment metrics should be incorporated to monitor algorithmic performance across different populations and subgroups, enabling timely identification and correction of potential inequities [168]. Additionally, technical approaches such as resampling, adversarial training, and domain adaptation can be used to reduce bias in model outputs.

### 7.2 Interpretability, transparency, and the “black box” in medical AI

In the evolution of medical AI, the concepts of “interpretability”, “transparency”, and the “black box” phenomenon are closely interrelated [169]. Interpretability refers to the extent to which an AI model can clearly explain to human users the reasons behind its predictions or decisions, enabling clinicians, patients, and regulators to understand the model’s rationale. Transparency emphasizes the openness and traceability of the system’s architecture, algorithmic details, and data processing workflows, allowing external experts or users to examine the inner operations [170], [171]. In contrast, the “black box” describes highly complex AI models, particularly DL networks, whose internal logic is largely unclear [172].

With the widespread adoption of DL and other complex models in medicine, model capacity and predictive accuracy have improved markedly. However, this progress has also resulted in a pronounced “black box” effect, reducing both interpretability and transparency. The black box phenomenon directly impacts the clinical acceptance and trustworthiness of medical AI. Clinicians and patients are often more concerned with understanding why a model produces a specific diagnosis or recommendation, not just the outcome itself. If a medical AI cannot justify its decisions, clinicians may be reluctant to use it, especially when predictions conflict with established medical knowledge or influence critical clinical decisions [173], [174].

To overcome these barriers, researchers are increasingly incorporating explainable AI (XAI) frameworks into medical model design [175]. By utilizing additive explanation algorithms such as SHAP, it is possible to attribute each prediction to specific input features in real time, identifying the most influential variables that shaped the model’s output [172]. This allows clinicians to know the model’s logic, supervise the decision-making process, and make informed judgments.

Improving transparency also involves open-sourcing algorithm code, fully disclosing model training procedures, data processing details, and decision thresholds, and continuously assessing model performance and bias in clinical practice [176], [177]. Only through these measures can AI systems escape the constraints of the “black box”, build trust among clinicians and patients, and achieve standardized application in healthcare settings.

### 7.3 Data security and privacy in medical AI

In the current era of deep integration between AI and healthcare, access to real patient data has become the foundation for training and validating medical AI models. Whether for automated disease diagnosis, treatment recommendation, or health risk prediction, high-quality and diverse clinical datasets are crucial for continuous model improvement [178]. To maximize generalizability and clinical applicability, data sharing across multiple institutions is often required [179]. However, this process inevitably raises significant concerns regarding patient privacy and data security. Patient data breaches can lead to a range of security risks and erode public confidence in both healthcare systems and emerging AI technologies [180]. This loss of trust may ultimately hinder the practical implementation of AI in clinical settings.

Researchers have developed multiple technical solutions to address these concerns. For end-to-end privacy protection, the PriMIA framework integrates differential privacy, encrypted aggregation, and secure multiparty computation, ensuring that data remains local throughout the model training process [181]. This effectively guards against re-identification attacks and model theft, while maintaining control over proprietary models, providing a robust security foundation for collaborative, multi-institutional medical AI. In the context of medical imaging data sharing, “digital masking” technology leverages 3D reconstruction and DL to permanently remove facial biometric features, retaining only disease-relevant information [182]. This greatly reduces the risk of identity leakage, increases patient acceptance of data sharing, and offers a novel paradigm for the compliant use of medical imaging data. Federated learning, as a leading approach for distributed collaborative training, allows models to be trained locally at each institution with only model parameters exchanged, never raw data. This enables diverse data sharing across regions and institutions while effectively protecting privacy, significantly improving model fairness, robustness, and generalizability, and supporting the establishment of multicenter medical AI systems [183], [184].

### 7.4 Legal and regulatory issues in medical AI

With the widespread application of AI in healthcare, ensuring its safe, effective, and compliant integration remains a critical challenge. Currently, there is no unified standard for the approval systems and legal frameworks for medical AI across different countries and regions, which complicates global implementation and dissemination. The European Union’s AI Act is the world’s first comprehensive regulatory framework for AI technology, specifically emphasizing high-risk categories for medical AI applications [185]. It mandates rigorous approval processes for AI systems involved in life and health, ensuring their transparency, safety, and compliance. For the shortcomings, AI Act simplifies “trustworthiness” into “risk acceptability,” relying on expert conformity assessments to determine whether AI systems can be deployed. However, this technocratic approach neglects the public’s actual perception of trust [186].

The General Data Protection Regulation (GDPR) imposes strict requirements for data privacy in AI applications, particularly in healthcare. GDPR emphasizes informed consent, data minimization, data security, and traceability of data [187]. This means that AI systems processing patient health data must ensure transparency and privacy protection while requiring organizations to comply with regulations during data collection, storage, processing, and sharing. GDPR presents a challenge for cross-border AI applications, as it restricts the free flow of patient data, which is critical for global data sharing in medical AI applications [188].

In the United States, the FDA regulates AI in medical devices typically through the Software as a Medical Device (SaMD) pathway, which requires AI models to demonstrate their accuracy, reliability, and clinical effectiveness [189]. However, due to the “continuous learning” nature of AI models, the FDA faces challenges in addressing dynamic algorithm changes, and existing approval processes may not be well-suited to the rapid development of AI technology. Additionally, data privacy laws such as Health Insurance Portability and Accountability Act (HIPAA) impose strict requirements on AI applications in healthcare, particularly regarding the protection and security of patient data [190].

Evaluating medical AI goes beyond assessing algorithmic performance. It also requires considering clinical effectiveness, representativeness of data sources, fairness, interpretability, and compatibility with existing healthcare systems [191]. However, there is no unified standard for evaluating the comprehensive performance of AI systems, resulting in significant variations in evaluation outcomes across regions and institutions, further complicating cross-national and cross-regional applications.

Therefore, the future should focus on promoting a globally unified AI evaluation standard, particularly in areas such as data privacy protection, algorithm interpretability, and fairness assessments. This will ensure that medical AI can be effectively applied within different legal and regulatory frameworks and truly benefit global patients.

## 8 Prospects and future directions

To improve the performance and efficiency of medical AI, it is essential to recognize that even the slightest error or “hallucination” in a clinical context can deprive patients of life-saving opportunities or even cause medical accidents [192]. Therefore, future medical AI model optimization should not focus solely on performance metrics, but also safeguard the fundamental principle of “safety and reliability,” prioritizing predictive accuracy and risk control. Medical AI should adopt a more conservative approach. In the face of uncertainty, the system should defer to human intervention rather than issue unreliable conclusions. This strategy helps minimize medical error rates, manage potential risks, and uphold patient safety.

The widespread adoption of medical AI in clinical practice is contingent upon the standardization of medical data sources [8]. Establishing high-quality standards for medical data is essential. These standards include strictly regulating data collection, annotation, storage, and sharing procedures, as well as improving representativeness and accuracy [193]. Only on the foundation of standardized and regulated data can medical AI models adapt to diverse clinical scenarios and achieve greater generalizability and fairness.

Furthermore, ensuring model transparency is essential [173]. Medical AI systems must avoid functioning as inscrutable “black boxes”; each stage of model reasoning should be both traceable and interpretable, thereby enabling clinicians to review, interrogate, and, when necessary, revise the system’s outputs. Integrating explainable algorithms and developing transparent, logical, and causally sound reasoning processes can help strengthen physician and patient trust in medical AI. Meanwhile, the industry should accelerate the standardization and disclosure of model evaluation criteria. It is essential to establish unified and scientific performance assessment systems that consider accuracy, safety, generalizability, fairness, and interpretability. Such standards are critical to ensuring the clinical reliability of medical AI.

Despite the rapid development of medical AI, it should neither replace nor attempt to replace professional clinicians at present. AI is better suited to serve as a supportive tool for physicians, particularly in tasks that are highly repetitive, workflow-driven, or data-intensive, reducing workload and improving efficiency and accuracy [194]. Numerous studies show that human-AI collaborative diagnostic strategies consistently outperform either human experts or AI models alone [195], [196]. By leveraging their complementary strengths, diagnostic performance and patient outcomes are significantly enhanced. Therefore, the future design of medical AI should emphasize deep integration with clinical teams, enabling “human-machine co-management” and collaborative decision-making to maximize the efficiency of medical resource utilization.

Moreover, the development of medical AI should progress beyond single-task functionalities toward fully integrated, multimodal, and end-to-end intelligent systems. Ideally, medical AI models will combine the interactive capabilities of LLMs for natural dialogue with patients, the analytical power of computer vision for interpreting medical images and pathology slides, and the capacity for evidence-based diagnostic reasoning and personalized treatment planning. In surgical settings, AI may even assist with certain procedures, reducing clinician burden and enhancing both safety and precision. In the longer term, the introduction of digital models, such as virtual organs, is expected to further innovate the virtual medical system [197], [198]. These high-fidelity digital biological models will enable us to realize risk-free personalized disease simulation and treatment prediction in the computer before any physical intervention.

In conclusion, medical AI represents a next-generation productivity tool with substantial transformative potential. It offers solutions to enduring challenges such as inequitable healthcare access, facilitates the implementation of precision medicine and intelligent health strategies, and supports the provision of comprehensive care for diverse populations, positioning itself as a central driver of global medical advancement. Achieving this goal requires that AI systems are always committed to protecting patient safety and clinical value. It also requires continuous improvement in model performance and ongoing strengthening of regulatory and ethical frameworks. Medical AI can be effectively and safely integrated into mainstream clinical practice only through adherence to these principles, thereby advancing human health in a more intelligent and equitable manner.

## Abbreviations

3D: Three-dimensional

AI: Artificial intelligence

EHRs: Electronic health records

FDA: U.S. food and drug administration

GDPR: General data protection regulation

GPT: Generative pre-trained transformer

LMICs: Low- and middle-income countries

LLMs: Large language models

M3FM: Medical multimodal multitask foundation model

MedLLM: Medical large language models

ML: Machine learning

NLP: Natural language processing

## Ethics approval and consent to participate

Not applicable.

## Funding

This work was supported by the National Natural Science Foundation of China (82230071, 32471396, 82427809), the Shanghai Committee of Science and Technology (23141900600), the Shanghai Clinical Research Plan (SHDC2023CRT013), the Shanghai Municipal Demonstration Project for Innovative Medical Device Applications (23SHS05700), and the Young Elite Scientist Sponsorship Program by China Association for Science and Technology (YESS20230049).

## CRediT authorship contribution statement

LB, FXW, and XT conceptualized the review. CZ wrote and edited the manuscript; CZ, JLL, ND, and YH collected published papers. LB, JCS, and PRS revised the manuscript. The illustrations in this review were created with Biorender.com by CZ. LB and JCS provided funding support. All authors reviewed and approved the final version of the manuscript.

## Data Availability

Not applicable.
